# Circular RNAs in Blood Malignancies

**DOI:** 10.3389/fmolb.2020.00109

**Published:** 2020-06-26

**Authors:** Olivia Perez de Acha, Martina Rossi, Myriam Gorospe

**Affiliations:** Laboratory of Genetics and Genomics, National Institute on Aging Intramural Research Program, National Institutes of Health, Baltimore, MD, United States

**Keywords:** circular RNAs, blood malignancies, leukemias, lymphomas, non-coding RNA

## Abstract

Circular (circ)RNAs influence a wide range of biological processes at least in part by interacting with proteins and microRNAs. CircRNAs expressed in the hematopoietic compartment have been increasingly recognized as modulators of physiological and pathological features of hematopoetic stem cell (HSC)-derived populations. In particular, several circRNAs were found to enhance or suppress tumor progression in blood malignancies such as leukemias and lymphomas. Moreover, numerous circRNAs have been proposed to help confer resistance to the conventional treatments used in hematopoietic cancers. Here, we review the most important circRNAs described thus far in acute myeloid leukemia (AML), chronic myeloid leukemia (CML), acute lymphoblastic leukemia (ALL), chronic lymphocytic leukemia (CLL), lymphomas, and multiple myeloma (MM). We discuss the usefulness of circRNAs as diagnostic and prognostic markers and their potential value as therapeutic targets.

## Introduction

The ENCyclopedia Of DNA Elements (ENCODE) project revealed that only 2% of the human genome encodes proteins (ENCODE Project Consortium, 2012). Subsequently, there has been a growing interest in studying RNAs without apparent coding function, collectively named non-coding RNAs (ncRNAs). Several ncRNAs, such as ribosomal (r)RNAs, transfer (t)RNAs, small nuclear (sn)RNAs, and small nucleolar (sno)RNAs are well-known for their role in “housekeeping” cell functions (Zhang et al., 2019). In addition, ncRNAs include heterogeneous transcripts with lesser-known regulatory functions, including microRNAs (miRNAs), piwi-interacting (pi)RNAs, small interfering (si)RNAs, and long non-coding (lnc)RNAs (Zhang et al., 2019).

Circular (circ)RNAs are a vast class of ncRNAs with covalently closed ends and lengths between ~100 to thousands of nucleotides (Kristensen et al., 2019). Unlike lncRNAs, circRNAs are usually well conserved among different species. They mainly originate through a process called backsplicing, in which the 3' and 5' ends of a precursor RNA are cleaved and ligated by the splicing machinery. Given the lack of 5' and 3' ends, circRNAs are not degraded by RNA exonucleases and therefore they are quite stable. Most of the circRNAs described to-date originate from exonic sequences (Chen et al., 2015), however they may also contain introns and intergenic sequences. CircRNAs can be localized in both the cytoplasm and the nucleus, as well as outside the cell in extracellular vesicles (Li et al., 2015). They often display a tissue-specific distribution and their expression may be altered in cancer and other pathologies (Kristensen et al., 2019; Liu et al., 2019; Mei et al., 2019; Vo et al., 2019). A growing body of evidence suggests that circRNAs play a role as potential prognostic and diagnostic biomarkers in cancer, given their high stability and specific expression patterns. CircRNAs are present in human body fluids like blood, urine, and saliva, and thus could be easily detected through non-invasive biopsies (Wang et al., 2018; Su et al., 2019; Verduci et al., 2019).

**Figure 1:**
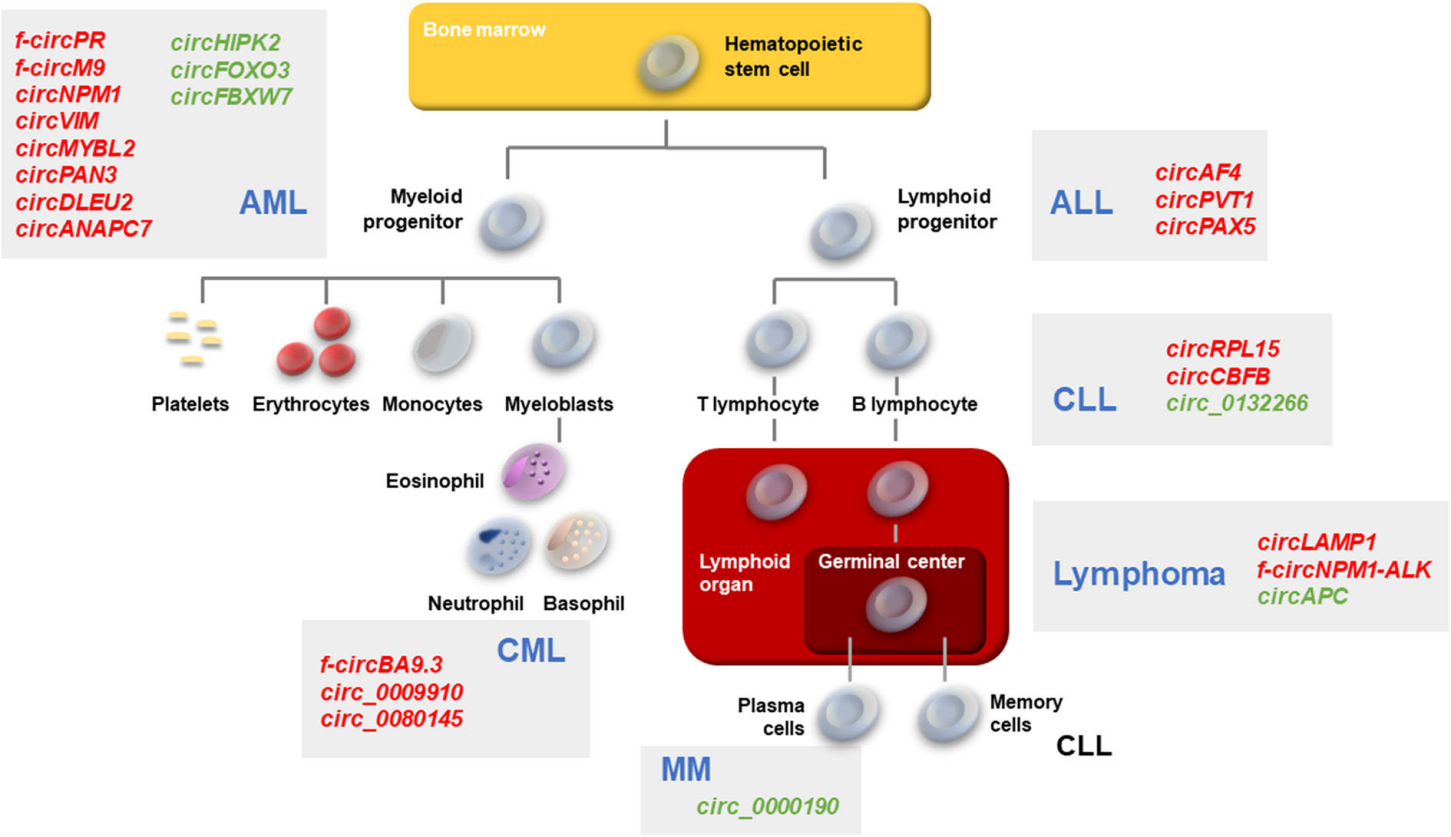
Schematic of hematopoiesis depicting the developmental cell types giving rise to the major leukemias and lymphomas. AML, CML, ALL, CLL, Lymphomas, and MM described in the text are represented. Gray boxes, the main circRNAs associated with each malignancy are indicated in red (upregulated in malignancy) or green (downregulated in malignancy).

A number of functions have been described for circRNAs, including synthesis of short polypeptides, recruitment of proteins to DNA, and scaffolding between specific enzymes and substrates (D'Ambra et al., 2019; Kristensen et al., 2019; Lei et al., 2020). Importantly, many circRNAs have been proposed to act as a “sponge” or decoy for microRNAs (miRNAs) and RNA-binding proteins (RBPs) (D'Ambra et al., 2019; Jamal et al., 2019; Kristensen et al., 2019; Verduci et al., 2019). CircRNAs bind and thereby functionally “neutralize” or inactivate miRNAs, thus restoring the translation of proteins that are otherwise suppressed by specific miRNA-mRNA binding events. This process is particularly effective when a circRNA is highly abundant and contains several binding sites for a target miRNA. For example, the well-known circRNA *CDR1as* (also called *ciRS-7*) has >60 binding sites for miR-7 (Hansen et al., 2013). The sponging of miR-7 by *CDR1as* has been described in Parkinson's disease, Alzheimer's disease, and several cancers (Shao and Chen, 2016). CircRNAs can also bind RBPs and these interactions may interfere with or enhance the functions of several proteins and mRNAs. For example, *circPABPN1* suppressed cell proliferation by interacting with the RBP HuR, preventing HuR from binding to *PABPN1* mRNA, and thereby suppressing the translation of PABPN1, a protein critically involved in cell proliferation (Abdelmohsen et al., 2017). Here, we review the increasingly recognized roles of circRNAs in hematological malignancies (Bonizzato et al., 2016; Mei et al., 2019), with a particular focus on the binding and possible sponging of oncogenic or tumor-suppressive miRNAs. These circRNAs, their effectors, and impacts on hematologic diseases are summarized in Table 1 and Figure 1.

**Table 1:** Circular RNAs implicated in hematological malignancies.

	**circRNA**	**Levels**	**miRNAs, RBPs, and pathways targeted**	**Impact on hematologic disease**	**References**
**ACUTE MYELOID LEUKEMIA (AML)**					
*PML-RARA*	***f-circPR (PML)***	*de novo*	Signaling through AKT	Increased cell proliferation Chemotherapy resistance	Guarnerio et al., 2016
*MLL-AF9*	***f-circM9***	*de novo*	Signaling through MAPK and AKT	Increased cell proliferation Chemotherapy resistance	Guarnerio et al., 2016
*NPM1*	***circNPM1 hsa_circ_0075001***	UP	miR-181 TLR signaling	Altered differentiation Promotion of leukemogenesis	Hirsch et al., 2017
*DLEU2*	***circDLEU2 hsa_circ_0000488***	UP	miR-496 and PRKACB	Increased cell proliferation Inhibition of apoptosis	Wu et al., 2018
*KLHL8*	***circKLHL8***	Associated with outcome	miR-155 and increased CDKN1, CDKN2, BCL6, TLR4, CEBP	Positive prognostic marker	Papaioannou et al., 2020
*FBXW7*	***circFBXW7***	DOWN	Signal transduction Leukocyte differentiation	Tumor suppression	Papaioannou et al., 2020
*FOXO3*	***circFOXO3***	DOWN	Apoptotic pathways	Induced apoptosis Diagnostic and prognostic biomarker	Zhou et al., 2019
*ANAPC7*	***circANAPC7 hsa_circRNA_101141***	UP	miR-181	Possible role in HSCs differentiation	Chen et al., 2018
*MFN2*	***circ_0009910 hsa_circRNA_100053***	UP	miR-20a-5p Proliferative pathways	Cancer growth	Ping et al., 2019a
*SLC30A7*	***circ_100290***	UP	miR-203/Rab10	Increased cell proliferation Inhibition of apoptosis	Fan et al., 2018
*WDR7*	***hsa_cir_0004277***	DOWN	Unconfirmed	Diagnostic and prognostic biomarker	Li et al., 2017
*(Unknown)*	***circ-0004136***	UP	miR-142, miR-29a	Increased cell proliferation Inhibition of apoptosis	Yuan et al., 2019
*VIMENTIN*	***circVIM***	UP	Unknown	Prognostic biomarker	Yi et al., 2019
*HIPK2*	***circHIPK2 (PML)***	DOWN	miR-124-3p CEBPA	Prognostic Biomarker Possible role in differentiation induced by ATRA treatment	Li et al., 2018a
*(Unknown)*	***hsa_circ_0004520***	UP	PLXNB2, VEGFA	Angiogenesis Prognostic biomarker	Lv et al., 2018
*MYBL2*	***circMYBL2 hsa_circ_0006332***	UP	Enhances FLT3 translation	Increased cell proliferation and chemoresistance Inhibition of apoptosis	Sun et al., 2019
*PAN3*	***circPAN3***	UP	miR-153-5p, miR-183-5p XIAP	Inhibitor of apoptosis Increased chemoresistance	Shang et al., 2019
**CHRONIC MYELOID LEUKEMIA (CML)**					
*BCR-ABL1*	***f-circBA9.3***	*de novo*	Apoptotic pathways	Negative prognostic factor Increase chemoresistance	Pan et al., 2018
*MFN2*	***circ_0009910 hsa_circRNA_100053***	UP	Unconfirmed in CML	Negative prognostic biomarker Imatinib resistance	Ping et al., 2019b
*(Unknown)*	***hsa_circ_0080145***	UP	miR-29b	Increased cell proliferation Inhibition of apoptosis	Liu et al., 2018
**ACUTE LYMPHOID LEUKEMIA (ALL)**					
*AF4*	***circAF4***	UP	miR-128-3p MLL-AF4	Promotes leukemogenesis *in vitro* and *in vivo*	Huang et al., 2019
*PVT1*	***circPVT1***	UP	miR-125, let-7	Increased cell proliferation Inhibition of apoptosis	Hu et al., 2018
*PAX5*	***circPAX5***	UP	miR-124 (unconfirmed)	May promote B cell maturation in pediatric patients	Gaffo et al., 2019
*HIPK3*	***circHIPK3***	UP	miR-124 (unconfirmed)	Unknown	Gaffo et al., 2019
*ENL, AF6, AF9, AF10, GAS7*	***circENL, circAF6, circAF9, circAF10, circGAS7***	Unknown	Unknown	Unknown	Huang et al., 2019
**CHRONIC LYMPHOID LEUKEMIA (CLL)**					
*RPL15*	***circRPL15 hsa_circ_0064574***	UP	miR-146b-3p RAF1	Increased cell proliferation Diagnostic biomarker	Wu et al., 2020
*CBFB*	***circCBFB hsa_circ_0000707***	UP	miR-607 FZD3, Wnt/β-catenin pathway activation	Increased cell proliferation Inhibition of apoptosis Prognostic and diagnostic marker	Xia et al., 2018
*MTO1*	***circ_0132266***	DOWN	miR-337-3p PML	Tumor suppressor	Wu et al., 2019
**LYMPHOMAS**					
*LAMP1*	***circLAMP1 hsa_circRNA_101303***	UP	miR-615-5p DDR2	Increased cell proliferation Inhibition of apoptosis	Deng et al., 2019
*APC*	***circAPC hsa_circ_0127621***	DOWN	miR-888 APC	Tumor suppressor Diagnostic and prognostic marker	Hu et al., 2019
*NPM1-ALK*	***f-circNPM1-ALK***	*de novo*	Unknown	Possible diagnostic marker for ALCL	Babin et al., 2018
**MULTIPLE MYELOMA (MM)**					
*Unknown*	***circ_0000190***	DOWN	miR-767-5p MAPK4	Prognostic marker Tumor suppressor Potential therapeutic target	Feng et al., 2019

### circRNAs in AML

Acute myeloid leukemia (AML) is the most common acute leukemia in adults, with an incidence of over 20,000 cases per year in the United States (De Kouchkovsky and Abdul-Hay, 2016). AML is characterized by the rapid growth of abnormal and immature white blood cells, inhibiting the production of normal hematopoietic cells in the bone marrow.

Many cytogenetic abnormalities causing AML have been characterized and include the large chromosomal translocations t(8;21), t(15;17), and t(9;11), which create the fusion proteins RUNX1-RUNX1T1, PML-RARA, and MLL-AF9, respectively (De Kouchkovsky and Abdul-Hay, 2016). Using patient samples, Guarnerio et al. (2016) found that the rearrangement of chromosomes led to the biogenesis of fusion-circRNAs (f-circRNAs) and identified two tumor-promoting f-circRNAs, *f-circPR*, and *f-circM9*, derived from the fusion transcripts *PML-RARA* and *MLL-MLLT3* (AF9), respectively. These f-circRNAs enhanced cell proliferation and promoted leukemogenesis *in vivo* in mice when co-expressed with their oncogenic fusion protein counterparts. Furthermore, f-circRNAs contributed to therapy resistance by conferring protection from apoptosis during treatment with the chemotherapeutic drugs arsenic trioxide (ATO) and cytarabine (Ara-C).

Cytogenetically normal AML (CN-AML) is not associated with chromosomal aberrations but is characterized by heterogeneous gene mutations with therapeutic and prognostic implications. For instance, mutations in *FLT3-IDT* (internal tandem duplication in the fms-related tyrosine kinase 3 gene) are associated with a higher risk of relapse, whereas mutations in the chaperone nucleophosmin gene (*NPM1*) are associated with a favorable prognosis in the absence of other mutations (De Kouchkovsky and Abdul-Hay, 2016). Hirsch et al. (2017) identified several *NPM1*-derived-circRNAs in CN-AML cell lines carrying either the normal or the mutated *NPM1* gene. The circRNA *hsa_circ_0075001* was elevated in AML cells independently of the *NPM1* mutational status. The levels of *hsa_circ_0075001* were higher in a cohort of 46 patients with undifferentiated blasts and correlated negatively with the expression of genes involved in Toll-like receptor (TLR) signaling, which is implicated in hematopoietic cell differentiation (Nagai et al., 2006; Okamoto et al., 2009; Eriksson et al., 2017). Moreover, in patients with high *hsa_circ_0075001* levels, the abundance of miR-181 target genes was reduced; the authors linked these two observations by noting that *NPM1* mRNA has miR-181 binding sites and circRNAs derived from *NPM1* may sequester miR-181 (Hirsch et al., 2017). Importantly, miR-181 is a critical regulator of cellular differentiation and hematological malignancies (Su et al., 2015).

Another circRNA upregulated in CN-AML primary samples, *circDLEU2* promoted tumor formation *in vivo* in mice. High levels of *circDLEU2* were proposed to reduce miR-496 function and to promote the expression of PRKACB (Protein Kinase, cAMP-dependent Catalytic β), leading to alterations in cell proliferation and apoptosis (Wu et al., 2018). In CN-AML patients, high levels of another circRNA, *circKLHL8*, correlated with better overall outcomes, and event-free survival, together with a lower percentage of malignant blasts in blood and bone marrow (Papaioannou et al., 2020). Two other circRNAs, *circFOXO3* and *circFBXW7*, were hypothesized to function as tumor suppressors in AML (Zhou et al., 2019; Papaioannou et al., 2020).

In recent years, additional circRNAs dysregulated in AML have been identified. Chen et al. (2018) found that *circANAPC7* (*circ_101141*) was upregulated in AML bone marrow samples and could play a role in the disease by sponging microRNAs in the miR-181 family, which regulate hematopoietic differentiation. Additional studies were performed on AML bone marrow samples; Ping et al. (2019a) identified *circ_0009910* as a circRNA that sponged the tumor-suppressor microRNA miR-20a-5p, thus promoting cancer growth, while Fan et al. (2018) reported that *circ_100290* promoted AML cell proliferation and inhibited apoptosis by sponging miR-293, ultimately increasing the expression of Rab10, a member of the oncogenic RAS family. Li et al. (2017) reported the dynamic expression of *circ_0004277* in AML patients: *circ_0004277* levels were low in newly diagnosed patients compared to healthy controls, but its expression was restored after complete response to induction therapy. Follow-up studies revealed that *circ_0004277* levels decreased again during relapse, confirming its potential value as a diagnostic and prognostic biomarker. Bioinformatic analysis predicted that *circ_0004277* might be part of a complex network including several miRNAs and mRNAs.

Yuan et al. (2019) reported a rise in *circ_0004136* in a pediatric AML cohort and proposed that *circ_0004136* promoted cell proliferation at least in part by binding and repressing miR-142, a microRNA known to be associated with pediatric AML. In a cohort of 113 AML patients, Yi et al. (2019) identified *circVIM*, derived from the *VIM* (vimentin) gene. Vimentin expression is known to be linked to tumor progression and can be a useful marker of the aggressiveness of certain cancers, such as gastric cancer (Fuyuhiro et al., 2010). Similarly, high levels of *circVIM* in AML were associated with shorter overall survival and leukemia-free survival, pointing to *circVIM* as a possible prognostic marker in AML (Yi et al., 2019).

Acute promyelocytic leukemia (APL), a less common form of AML, is characterized by the formation of the promyelocytic leukemia/retinoic acid receptor α (PML/RARα) fusion protein, which causes many of the features of the disease. APL has a high responsiveness to all-*trans* retinoic acid (ATRA) treatment, which leads to further differentiation and maturation of the leukemic cells (Cicconi et al., 2018; Li et al., 2018a). (Li et al., 2018a) identified several circRNAs differentially expressed in APL-derived NB4 cells during ATRA treatment and validated in patient samples: *circHIPK2, circHIPK3, circPVT1, circRELL1*, and *circSMARCA5*. The authors focused on the levels of *circHIPK2*, which decline in newly diagnosed persons and are restored after complete remission. Further experiments proved a key relationship between this circRNA and cell maturation: *circHIPK2* sponged miR-124-3p and increased the expression of CEBPA, a transcription factor involved in hematopoiesis. Altogether, the results highlighted *circHIPK2* as a potential biomarker for APL.

Extramedullary Infiltration (EMI) is a poor prognostic indicator in AML characterized by the accumulation of blasts in extramedullary locations, including spleen, liver, skin, and central nervous system. Lv et al. (2018) found that most circRNAs upregulated in EMI bone marrow samples might be proposed to influence cell adhesion, migration, and signal transduction. Among them, *hsa_circ_0004520* is predicted to modulate the expression of VEGFA (vascular endothelial growth factor A), which could contribute to angiogenesis in AML-EMI.

The complexity of molecular and cytogenetic abnormalities is a challenge for the design of AML therapy. While induction therapy with cytarabine and an anthracycline remains a standard of care in AML, resistance may develop over time through different mechanisms (Dombret and Gardin, 2016). Specific tyrosine kinase inhibitors (TKI) have been developed to treat the aforementioned FLT3-ITD AML, but despite success in achieving remission in clinical trials, patients often relapsed or acquired resistance over time (De Kouchkovsky and Abdul-Hay, 2016; Sun et al., 2019). Sun et al. (2019) reported that *circMYBL2* silencing restored sensitivity of human FLT3-ITD^+^ cells to the TKI quizartinib and inhibited cell proliferation in culture as well as in mice; mechanistically, *circMYBL2* enhanced FLT3 translation by facilitating the binding between *FLT3* mRNA and the RBP polypyrimidine tract-binding protein 1 (PTBP1).

Shang et al. (2019) identified *circPAN3* as a key factor in doxorubicin resistance in AML cell lines (33). *CircPAN3* was shown to bind miR-153-3p and miR-183-5p, in turn modulating the expression levels of XIAP (X-linked inhibitor of apoptosis protein), a key protein implicated in autophagy and apoptosis. In addition, *circPAN3* downregulation restored drug sensitivity, suggesting a role for this circRNA in AML resistance to conventional chemotherapies.

### circRNAs in CML

Chronic myeloid leukemia (CML) is a rare clonal myeloproliferative malignancy with an annual incidence of one to two cases per 100,000 persons (Zhou and Xu, 2015). The cytogenetic hallmark of CML is the Philadelphia (Ph) chromosome, generated by the reciprocal translocation between the long arms of chromosomes 9 and 22, t(9;22) (q34;q11). The fusion gene *BCR-ABL1* arises as a consequence of the translocation and leads to the production of the oncogenic fusion protein BCR-ABL1, a tyrosine kinase that induces the phosphorylation, activation, and dysregulation of signaling molecules involved in the survival and growth of bone marrow progenitor cells (Kang et al., 2016). TKIs are the standard choice of treatment for CML, but their effectiveness depends on the phase of the disease and is challenged by the development of drug resistance over time (Litwinska and Machalinski, 2017; Patel et al., 2017). High levels of BCR-ABL1 kinase activity may arise as a consequence of gene amplification and Ph duplication, and may be sufficient to confer TKI resistance. In addition, specific mutations in the kinase domain (KD) of BCR-ABL1 may enhance drug resistance (Soverini et al., 2014; Patel et al., 2017).

Pan et al. (2018) identified f-circRNA *circBA9.3*, derived from the *BCR-ABL1* mRNA, as potentially involved in drug resistance to Imatinib, a second-generation TKI. *CircBA9.3* expression was higher in TKI-resistant patients compared to responsive controls. Moreover, *circBA9.3* transfection in BCR-ABL-negative cell lines enhanced proliferation and cancer progression. Further experiments confirmed that *circBA9.3* levels were positively correlated with the expression of BCR-ABL1. Regardless of whether first or second generation TKIs were administered, cells overexpressing *circBA9.3* displayed less apoptosis compared to controls.

Further studies investigated the role of circRNAs in CML drug resistance. Ping et al. (2019b) used circRNA microarrays to assess circRNA profiles in CML and found that *hsa_circ_100053* levels increased in both cells and serum of CML patients. In keeping with the proposal that *hsa_circ_100053* might be a potential biomarker in CML, higher expression of *hsa_circ_100053* was associated with advanced clinical stage, BCR/ABL1 mutational status and resistance to Imatinib. High levels of *hsa_circ_100053* were suggested as a negative prognostic factor in the overall survival of CML patients.

Through an RNA-sequencing screen, Liu et al. (2018) identified *hsa_circ_0080145* as being upregulated in cells from CML patients and in cell lines K562 and KU812. Moreover, *hsa_circ_0080145* silencing suppressed leukemic cell proliferation. In functional experiments, the authors found that *hsa_circ_0080145* was capable of sponging miR-29b, and the targets of miR-29b were predicted with the tool miRTarBase (http://miRTarBase.mbc.nctu.edu.tw/), which contains interactions validated experimentally. Gene ontology analysis revealed that these genes belong to three main groups: systemic lupus erythematosus pathway, cAMP signaling pathway, and heterocycle biosynthetic process. In agreement with these findings, a previous study reported that miR-29b was downregulated in CML and overexpression of miR-29b in K562 cells inhibited leukemic cell growth and promoted apoptosis through regulation of the BCR-ABL1 tyrosine kinase (Li et al., 2013). In addition, several other oncogenes could be potentially silenced by miR-29, including the antiapoptotic protein MCL1, upstream inhibitors of p53, DNA methyltransferases, and extracellular matrix proteins (Li et al., 2013).

Together, these findings highlight the importance of circRNAs as potential CML biomarkers with key roles in drug resistance and as targets for new therapeutic treatments.

### circRNAs in ALL

Acute lymphoblastic leukemia (ALL) is the most common cancer among children in the USA and the most frequent cause of cancer death in young people (Hunger and Mullighan, 2015). ALL may arise from B-cell (B-ALL) or T-cell (T-ALL) precursors. Current medical treatments for ALL allow high survival rates, but relapse occurs in 15–20% of pediatric patients and is associated with a higher risk of treatment failure (Schrappe et al., 2012). Most somatic mutations acquired in ALL are chromosome rearrangements, such as translocations and hyperdiploidy. Translocation t(4;11)(q21;q23) resulting in the chimeric product MLL-AF4 is commonly identified in infant pro-B-ALL and has poor prognosis (Mrózek et al., 2009).

The first studies on circRNAs differentially expressed in ALL (Salzman et al., 2012) found that >10% of the transcripts encoded by hundreds of genes in naïve B cells (CD19+) and hematopoietic stem cells (CD34+) were circRNAs. Subsequently, Huang et al. (2019) identified different circRNAs derived from the MLL partner fusion gene *AF4*. The levels of *circAF4(ex3-4)* were higher in the leukemia cell line analyzed (RS4;11) and patients under 8 years of age. Moreover, *circAF4* levels correlated with the severity of disease, and *circAF4* silencing led to increased apoptosis in cells carrying the *MLL-AF4* translocation. In mice, *circAF4* knockdown improved survival and reduced spleen infiltration. By binding miR-128-3p, *circAF4* might sequester the microRNA away from the fusion *MLL-AF4* mRNA and enable MLL-AF4 expression. Simultaneous *circAF4* silencing and miR-128-3p overexpression *in vivo* supported this regulatory axis and suggested that *circAF4* acts as an oncogenic circRNA in leukemia. In a further study, Dal Molin et al. (2019) found that specific rearrangements leading to fusions between *MLL* and other genes not only generated alternative isoforms of circRNAs in different subtypes of leukemias, but may also contribute to the production of disease-associated aberrant circRNAs.

Hu et al. (2018) found that *circPVT1* dysregulation promoted cell proliferation and inhibited apoptosis in ALL cell lines by sponging miR-125 and let-7, ultimately increasing the expression of the oncogene MYC and the anti-apoptotic protein BCL2. Other studies showed that *circPVT1* regulated the function of let-7 family members which function as tumor suppressors (Panda et al., 2017a). *CircPVT1* had been previously described as a negative prognostic factor for gastric cancer (Chen et al., 2017a). In sum, by interfering with let-7 function, *circPVT1* was proposed to promote ALL leukemogenesis (Hu et al., 2018).

Gaffo et al. (2019) identified and quantified bioinformatically the circRNAs expressed in T cells, B cells and monocytes in physiological conditions; as expected, circRNA signatures varied with stage of differentiation and cell type. Further analysis of circRNAs differentially expressed in B-cell precursors of ALL pediatric patients found upregulated *circPVT1, circHIPK3*, and *circPAX5*. The *PAX5* (paired box protein five) gene encodes transcription factor BSAP (B-cell-lineage-specific activator protein), with a key role in defining and maintaining B-cell identity. The binding of *circPAX5* and *circHIPK3* to miR-124-5p was proposed to synergistically interfere with B cell maturation and promote disease progression.

### circRNAs in CLL

Chronic lymphocytic leukemia (CLL) is characterized by the accumulation of small and mature CD5^+^ B cells in blood, bone marrow and secondary lymphoid organs. CLL B-cells have a relatively low proliferation rate and high resistance to apoptosis (Kipps et al., 2017). The molecular classification of the disease relies on whether the neoplastic cells express a mutated or unmutated form of the immunoglobulin heavy chain variable region gene (*IGHV*), with the unmutated IGHV being a marker of poor prognosis and shorter survival. The variability in clinical behaviors reflects the underlying genetic heterogeneity (Kipps et al., 2017). To date, three main circRNAs have been studied in CLL: *circRPL15* (Wu et al., 2020), *circCBFB* (Xia et al., 2018), and *circ_0132266* (Wu et al., 2019).

*CircRPL15* was evaluated as a potential biomarker for the diagnostic screening in plasma from patients with CLL, especially in cases without IGHV mutation (Wu et al., 2020). Upregulated *circRPL15* was proposed to sponge miR-146b-3p, thereby increasing RAF1 protein levels. As an effector of the proliferative RAS pathway, RAF1 could in turn phosphorylate and thereby activate MAPK (mitogen-activated protein kinase) signaling, promoting cell growth. In support of this paradigm, knockdown of *circRPL15* in human cell lines reduced the phosphorylation of mitogenic factors (Wu et al., 2020) and an earlier study by Wang et al. (2008) found that RAF1 was overexpressed in CLL.

After finding that *circCBFB*, derived from *CBFB* (core-binding factor subunit beta) pre-mRNA, was upregulated in untreated CLL cells from patients, Xia et al. (2018) proposed that it could serve as a prognostic and diagnostic marker in CLL patients. Mechanistically, *circCBFB* was found to activate the Wnt/ß-catenin pathway in human cell lines by binding miR-607 and thereby derepressing production of Frizzled (FZD3), a receptor for Wnt; the ensuing proliferative and anti-apoptotic phenotype could therefore be involved in CLL progression. Moreover, higher levels of *circCBFB* and *circRPL15* correlated with poor overall survival and shorter survival time.

Wu et al. (2019) proposed a tumor-suppressor role for *circ_0132266* in CLL based on the ability of *circ_0132266* to bind miR-337-3p in CLL cell lines. PML (promyelocytic leukemia protein), a broad regulator of gene expression and cell viability, is a key target of miR-337-3p. The authors linked the reduction in *circ_0132266* levels to the increased miR-337-3p levels and proposed that by sponging miR-337-3p, *circ_0132266* might be tumor-suppressive in CLL.

### circRNAs in Lymphomas

Lymphomas originate from lymphocytes or their progenitor cells and tend to be localized in lymph nodes and the lymphatic system. Due to the high heterogeneity of lymphocytes, given the diverse lineages from which they arise, the stages of differentiation, and their specific functions, the classification of lymphomas is quite complex. Lymphomas are classified into Hodgkin and non-Hodgkin, the latter being more common; the non-Hodgkin's lymphoma can be divided into two major groups depending on the cell lineage: B-cell and T-cell lymphomas (Swerdlow et al., 2016).

In T-cell lymphoblastic lymphoma (T-LBL) cell lines, Deng et al. (2019) described a regulatory axis in which *circLAMP1* was proposed to sponge miR-615-5p, thereby increasing the levels of DDR2 (discoidin domain receptor tyrosine kinase 2). DDR2 encodes a member of the receptor tyrosine kinase (RTKs) protein family, is induced by collagen, and in turn activates signal transduction pathways involved in proliferation, extracellular matrix remodeling, wound repair, and tumor invasiveness. Gain- and loss-of function experiments supported a role for *circLAMP1* in promoting cell proliferation and repressing apoptosis in T-LBL cells.

Diffuse large B-cell lymphoma (DLBCL) is the most common type of non-Hodgkin lymphoma worldwide (Li et al., 2018b). Morphologically, it is characterized by large B cells arranged in a diffuse pattern. RNA-seq analysis of DLBCL identified several genetic abnormalities and different mutation profiles. At present, the standard of care received by DLBCL patients is based on a combination of a monoclonal antibody (rituximab) and chemotherapy, a regimen called R-CHOP. The current 5-year overall survival rate is estimated around 60% (Li and Medeiros, 2018). *CircAPC*, downregulated in DLBCL cell lines and plasma from patients (Hu et al., 2019), originates from the backsplicing of the linear adenomatous polyposis coli (*APC*) gene, encoding a major regulator of cell proliferation. The tumor suppressor functions of the protein APC include regulation of cell adhesion, inhibition of Wnt/β-catenin pathway and control of cell cycle progression (Zhang and Shay, 2017). Importantly, DLBCL usually affects the gastrointestinal tract, where APC expression is most prominent (Li et al., 2018b). *CircAPC* was detected in both the cytoplasm and the nucleus of DLBCL cells (Hu et al., 2019). In the nucleus, *circAPC* bound the APC promoter and recruited DNA demethylase Ten-Eleven Translocation 1 (TET1), thereby reducing methylation and increasing *APC* transcription. In the cytoplasm, *circAPC* sequestered miR-888, enhancing APC translation and eventually inhibiting the proliferative Wnt/β-catenin pathway. In short, reduced levels of *circAPC* were proposed to promote DLBCL progression. Further analysis pointed to *circAPC* as a diagnostic and prognostic tool; for instance, low *circAPC* levels correlated with resistance to R-CHOP treatment, low International Prognostic Index (IPI) and advanced staging (formulated with the Ann-Harbor staging classification). Ectopic *circAPC* expression was shown to suppress DLBCL proliferation *in vitro* and *in vivo* in a mouse xenograft model.

ALCL (Anaplastic Large Cell Lymphoma) is a rare but highly aggressive T-cell non-Hodgkin lymphoma that originates from a chromosomal translocation that produces the fusion protein NPM1-ALK, a constitutively active tyrosine kinase (Fuchs et al., 2019). Babin et al. (2018) employed the CRISPR/Cas9 technology to generate the same translocation in different mouse and human cell lines and identified novel *f-circRNAs*, specifically *f-circRNA-mA* and *f-circRNA-hD* (collectively called *f-circRNA-NPM1-ALK*). This pool of circRNAs was also identified in some of the original tumors, thus representing a possible diagnostic marker for ALCL.

### circRNAs in MM

Multiple myeloma (MM) is characterized by proliferation of immunoglobulin-secreting plasma cells within the bone marrow. It is the second most common hematological cancer, accounting for 10% of all blood malignancies (Walker et al., 2014), with a life expectancy ranging from a few months to >10 years and hampered by an extremely rate of metastasis and drug resistance (Palumbo et al., 2015). High-risk cytogenetic subgroups, including those with deletion of chromosome 17p or gain of chromosome 1q21, progress rapidly and are associated with shortened overall survival (Sherbenou et al., 2016).

Feng et al. (2019) found that *circ_0000190* was downregulated in bone marrow biopsies and peripheral blood derived from MM patients. The authors propose that the reduction in *circ_0000190* led to increased levels and function of miR-767-5p, which in turn lowered the levels of MAPK4, an inhibitor of MM progression. Analysis of 47 patients revealed that higher expression of *circ_0000190* was associated with longer progression-free disease and improved overall survival. In murine xenograft models, ectopic expression of *circ_0000190* reduced tumor progression by 60%, underscoring the potential therapeutic value of this circRNA. Interestingly, the tumor-suppressor role of *circ_0000190* was originally described in gastric cancer (Chen et al., 2017b).

## Closing Remarks and Challenges Ahead

In this review, we have highlighted the progress made in identifying circRNAs implicated in hematological malignancies. Although many functions have been proposed for circRNAs (Panda et al., 2017b), most of the examples to-date underscore their ability to bind other molecules and sequester them away from miRNAs and RBPs. Through these specific interactions, circRNAs may influence the levels of proteins under the control of such miRNAs and RBPs. As uncovered in this review, many of these target proteins are implicated in controlling cell proliferation and survival. In the context of hematological diseases, dysregulation of RBPs and miRNAs has been linked to the initiation and progression of malignancy, chemotherapy resistance, and poor clinical outcome. To apply the emerging knowledge of circRNAs as potential interventions in the progression and treatment of leukemias and lymphomas, we have identified several challenges.

First, superior methods must be developed to identify and quantify circRNAs in all systems, including hematologic malignancies. More complete databases with comprehensive circRNA annotations are needed, particularly those that include information on tissue-specific circRNAs. In this regard, normalization of circRNA nomenclature will be particularly helpful to improve clarity across studies. Molecular methodologies must also improve to offer more sensitive and specific means of detecting circRNAs with diagnostic and prognostic value, given that they are often in quite low abundance.

Second, the field must adopt more rigorous methods of elucidating circRNA function and circRNA-interacting molecules. While the notion of circRNAs as “microRNA sponges” is certainly attractive, few studies have included the careful stoichiometric measurements and molecular biology interventions to demonstrate that a circRNA is indeed a sponge for an interacting microRNA. These careful analyses are particularly important, since many circRNAs exist in low copy numbers (often one or less per cell) while microRNAs often exist in hundreds or more copies per cell, so the sponging model may not be correct in every case.

Third, hematopoietic malignancies are particularly well suited for RNA-directed therapies such as antisense oligomers (ASOs), given their immediate access in the blood. However, the fact that the vast majority of circRNAs share most of their sequence with the parent linear RNA challenges these efforts. Improved bioinformatic and molecular approaches to optimize the targeting of the junction sequences of circRNAs will enable more accurate targeting of circRNAs for elimination in therapeutic settings. Conversely, for circRNAs with beneficial effects in hematopoietic malignancies, ectopic delivery methods could have therapeutic advantage; such delivery methods also await extensive development and optimization for use in the clinic.

In closing, we are gaining tools and knowledge of circRNAs implicated in hematologic cancers. The expansion and refinement of these tools and knowledge in the near future will enable the development of effective means of diagnosis, prognosis, and therapy directed at circRNAs in leukemias, lymphomas, and other hematologic malignancies.

## Author Contributions

OP, MR, and MG wrote the paper.

## Conflict of Interest

The authors declare that the research was conducted in the absence of any commercial or financial relationships that could be construed as a potential conflict of interest.
